# Deep learning of longitudinal chest X-ray and clinical variables predicts duration on ventilator and mortality in COVID-19 patients

**DOI:** 10.1186/s12938-022-01045-z

**Published:** 2022-10-14

**Authors:** Hongyi Duanmu, Thomas Ren, Haifang Li, Neil Mehta, Adam J. Singer, Jeffrey M. Levsky, Michael L. Lipton, Tim Q. Duong

**Affiliations:** 1 Department of Radiology, Albert Einstein College of Medicine, Bronx, NY 10461 USA; 2 Department of Emergency Medicine, Renaissance School of Medicine, Stony Brook University, Stony Brook, NY 11794 USA; 3 Department of Radiology, Montefiore Medical Center, Albert Einstein College of Medicine, Bronx, NY 10461 USA

**Keywords:** Coronavirus, Deep learning, Chest X-ray, Mortality, Ventilation

## Abstract

**Objectives:**

To use deep learning of serial portable chest X-ray (pCXR) and clinical variables to predict mortality and duration on invasive mechanical ventilation (IMV) for Coronavirus disease 2019 (COVID-19) patients.

**Methods:**

This is a retrospective study. Serial pCXR and serial clinical variables were analyzed for data from day 1, day 5, day 1–3, day 3–5, or day 1–5 on IMV (110 IMV survivors and 76 IMV non-survivors). The outcome variables were duration on IMV and mortality. With fivefold cross-validation, the performance of the proposed deep learning system was evaluated by receiver operating characteristic (ROC) analysis and correlation analysis.

**Results:**

Predictive models using 5-consecutive-day data outperformed those using 3-consecutive-day and 1-day data. Prediction using data closer to the outcome was generally better (i.e., day 5 data performed better than day 1 data, and day 3–5 data performed better than day 1–3 data). Prediction performance was generally better for the combined pCXR and non-imaging clinical data than either alone. The combined pCXR and non-imaging data of 5 consecutive days predicted mortality with an accuracy of 85 ± 3.5% (95% confidence interval (CI)) and an area under the curve (AUC) of 0.87 ± 0.05 (95% CI) and predicted the duration needed to be on IMV to within 2.56 ± 0.21 (95% CI) days on the validation dataset.

**Conclusions:**

Deep learning of longitudinal pCXR and clinical data have the potential to accurately predict mortality and duration on IMV in COVID-19 patients. Longitudinal pCXR could have prognostic value if these findings can be validated in a large, multi-institutional cohort.

## Introduction

Since the first coronavirus disease 2019 (COVID-19) case was reported in December 2019 [1, 2], 500 million people have been infected and more than 6 million people have died worldwide (Jun, 2022) [3]. The role of chest imaging in the diagnosis, prognosis and treatment of this infectious disease has evolved over the course of the COVID-19 pandemic. During the initial outbreak in China when virus assays were unreliable, computed tomography (CT) of the lung was the primary diagnostic tool used for triage and diagnosis [4–7]. Portable chest X-ray (pCXR) [8–11] is currently widely used to evaluate the spatial extent and location of lung infection associated with COVID-19 because the widely available imaging equipment is portable, can be dedicated for use in patients with suspected infection, and can be readily disinfected between uses, avoiding cross-contamination. The hallmarks of COVID-19 lung infection on pCXR include bilateral or peripheral hazy opacities and airspace consolidation [12].

In principle, pCXR could also be used to monitor disease progression and treatment response, optimize mechanical ventilator settings, determine when to safely extubate, and predict clinical outcomes in COVID-19 patients. However, pCXR is not currently being used in this capacity and thus the potential of pCXR in COVID-19 is not yet fully realized. This is in part because the temporal progression of COVID-19 lung infection on pCXR is incompletely understood [11]. pCXR has become more relevant because a disproportionally large percentage of COVID-19 patients require invasive mechanical ventilation (IMV) for a longer duration than patients with other similar lung infections [13, 14]. Improved understanding of the temporal progression of COVID-19 lung infection on pCXR could be leveraged to address resource allocation in the event of a shortage of mechanical ventilators, as occurred during peak periods of COVID-19 in the United States and around the world [15].

Machine learning (ML), including deep learning, is increasingly being used in medicine, including radiology [16–18]. In contrast to conventional analysis methods, which specify the relationships among data elements to outcomes, ML employs computer algorithms to identify relationships among different data elements to inform outcomes without the need to specify such relationships a priori. ML can accurately estimate risk in the Framingham Risk Score for coronary heart disease [19] and detect lung nodules on pCXR [20] without a priori specification of the data elements that lead to the determination.

A few studies have explored the use of ML to predict mortality based upon pCXR at admission to the emergency room [21] and associate radiological pCXR scores with clinical outcomes [22–28]. Prediction of COVID-19 outcomes based on a single pCXR at admission, however, is likely inadequate [11]. There are no studies to date that use ML analysis of longitudinal pCXR to predict the duration of the need for IMV or mortality associated with COVID-19. ML is well-suited to address such COVID-19 outcome prediction problem because the temporal relationships of serial imaging characteristics and serial clinical variables with outcomes are complex and cannot be readily parameterized to predict eventual outcomes.

The goal of this study was to determine whether deep learning of longitudinal pCXR could accurately predict the duration of IMV (i.e., how much time the patient needs to be on IMV) and in-hospital mortality in COVID-19 patients, as predicting mortality or the duration IMV using data from a single time point at admission is likely suboptimal. We compared prediction performance using a single time point against serial pCXR. We further considered a large array of non-imaging variables (such as demographic, comorbidity, serial vital signs and serial laboratory tests) to improve the predictive models. Deep learning of serial imaging and non-imaging clinical data has the potential to better inform the management of COVID-19 patients in time-sensitive, stressful, and potentially resource-constrained environments.

## Results

**Fig. 1:**
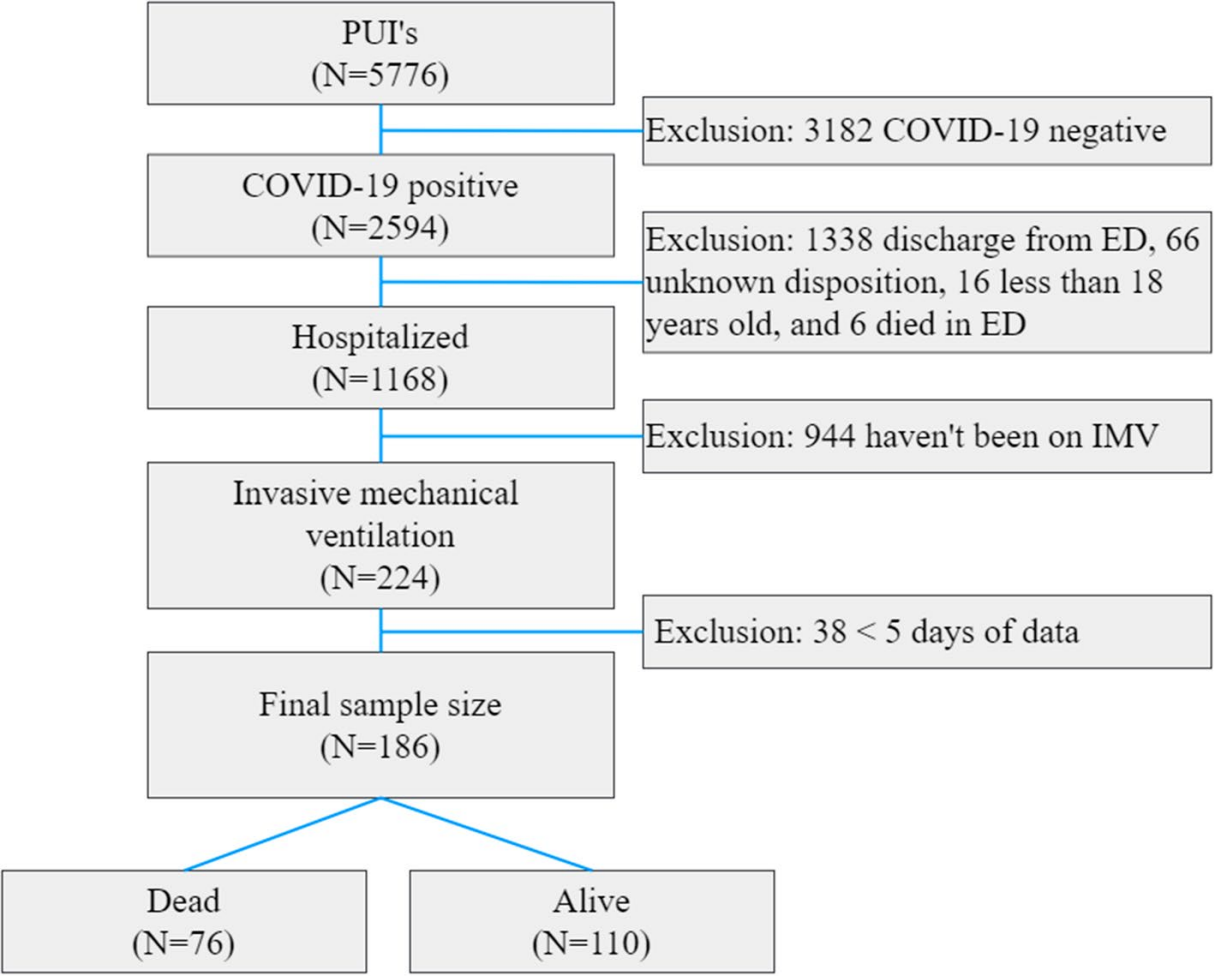
Patient selection flowchart

Figure 1 shows the flowchart of patient selection. At the time of this study, our registry of patients presenting to the emergency department (ED) with suspected COVID-19 (otherwise known as persons under investigation) consisted of 5,766 patients from February 7, 2020, to Jun 30, 2020. A subset of clinical variables using various analysis methods in this cohort had previously been published but addressing completely different questions [29, 30]. Only patients who were diagnosed with COVID-19 by real-time polymerase chain reaction (RT-PCR) for severe acute respiratory syndrome coronavirus 2 (SARS-CoV-2) were included in the study. Inclusion criteria were SARS-CoV-2-positive patients requiring IMV. Patients younger than 18 years of age were excluded. To maintain the same cohort that has at least 5 consecutive day data, patients with less than 5-day data were excluded. The final sample size after exclusions consisted of 110 IMV survivors and 76 IMV non-survivors prior to discharge.

Table 1 summarizes the demographics and comorbidities, as well as the laboratory tests and vital signs, stratified by primary outcome (110 IMV survivors, 76 IMV non-survivors). Patients who expired were older than those who survived (median age: 67 years [IQR: 58,73] versus 56 years [IQR: 50,64], *p* < 0.001). With the exception of smoking history, hypertension, and coronary artery disease, none of the comorbid conditions were considered significantly different between groups (*p* > 0.05). Other than ALT and HCO_3_, all blood markers and all vital signs differed between groups.

**Table 1:** Patient demographics, comorbidities, and clinical variables of dead and alive patients

	Patients, no. (%)		
	Died (*n* = 76)	Survived (n = 110)	*p* value
Demographics			
Age, median (IQR)	67 (58, 73)	56 (50, 64)	< 0.001
Sex			0.117
Male	59 (77.6%)	74 (67.3%)	
Female	17 (22.3%)	36 (32.7%)	
Ethnicity			0.374
Hispanic/Latino	20 (26.3%)	35 (31.8%)	
Non-Hispanic/Latino	44 (57.9%)	61 (55.5%)	
Unknown	12 (15.8%)	14 (12.7%)	
Comorbidities			
Smoking history			0.036
Current smoker	4 (5.3%)	3 (2.7%)	
Former smoker	21 (27.6%)	14 (12.7%)	
Never smoker	46 (60.5%)	86 (78.2%)	
Unknown	5 (6.6%)	7 (6.4%)	
Diabetes	25 (32.9%)	32 (29.1%)	0.585
Hypertension	46 (60.5%)	45 (40.9%)	0.008
Asthma	7 (9.2%)	12 (10.9%)	0.705
COPD	7 (9.2%)	6 (5.5%)	0.348
Coronary artery disease	18 (23.7%)	8 (7.3%)	0.004
Heart failure	6 (7.9%)	3 (2.7%)	0.141
Cancer	3 (3.9%)	3 (2.7%)	0.656
Immunosuppression	2 (2.6%)	9 (8.2%)	0.086
Chronic kidney disease	5 (6.6%)	6 (5.5%)	0.755
Laboratory findings at admission, median (IQR)			
Alanine aminotransferase, U/L (alt)	43 (24, 71)	43 (25, 80)	0.194
C-reactive protein, mg/dL (crp)	10.6 (4.9, 19.5)	5.3 (1.6, 12.1)	< 0.001
D-dimer, ng/mL (ddim)	1574 (793, 3290)	887 (498, 1894)	< 0.001
Ferritin, ng/mL (fer)	1267 (776, 2149)	861 (478, 1432)	< 0.001
Lactate dehydrogenase, U/L (ldh)	540 (411, 696)	392.0 (298, 512)	< 0.001
White blood cells, × 10^3^/ml (wbc)	13.0 (8.9, 19.3)	10.9 (8.4, 14.3)	< 0.001
Lymphocytes, % (lym)	4.5 (2.1, 8.0)	8.9 (4.5, 15.0)	< 0.001
Procalcitonin, ng/mL (procal)	0.7 (0.3, 1.9)	0.2 (0.1, 0.6)	0.019
Troponin T, ng/mL (tnt)	0.0 (0.0, 0.1)	0.0 (0.0, 0.0)	< 0.001
Aspartate aminotransferase, U/L (ast)	49.0 (32.0, 76.0)	38.0 (25.0, 63.0)	0.005
Creatinine, mg/dL (crt)	1.4 (0.9, 2.7)	0.8 (0.6, 1.3)	< 0.001
Blood gases and others			
pCO_2_	48.0 (42.0, 57.0)	47.0 (40.0, 53.0)	0.020
HCO_3_	26.0 (22.0, 31.0)	26.8 (23.0, 31.0)	0.113
pH	7.3 (7.3, 7.4)	7.4 (7.3, 7.4)	< 0.001
pO_2_	78.0 (64.9, 99.0)	82.7 (69.0, 105.0)	0.001
Hematocrit (hcrit)	31.2 (26.7, 37.0)	31.7 (27.4, 37.7)	< 0.001
Potassium, mEq/L (k)	4.3 (3.9, 4.9)	4.1 (3.7, 4.5)	< 0.001
Sodium, mEq/L (Na)	141.0 (137.0, 147.0)	141.0 (138.0, 145.0)	< 0.001
Vital signs, median (IQR)			
Heart Rate, bpm (hr)	86.0 (72.0, 101.0)	84.0 (71.0, 97.0)	< 0.001
Respiratory rate, bpm (rr)	25.0 (20.0, 30.0)	23.0 (20.0, 27.0)	< 0.001
Oxygen saturation (o2)	96.0 (93.0, 98.0)	97.0 (94.0, 99.0)	< 0.001
Systolic blood pressure, mmHg (sbp)	122.0 (109.0, 138.0)	124.0 (111.0, 142.0)	0.008
Diastolic blood pressure, mmHg (dbp)	64.0 (58.0, 72.0)	67.0 (60.0, 76.0)	0.005
Mean arterial pressure, mmHg (map)	84.0 (77.0, 95.0)	88.0 (79.0, 98.0)	< 0.001
Temperature, °C (temp)	37.0 (36.7, 37.5)	36.9 (36.7, 37.2)	< 0.001
FiO2, %	70.0 (50.0, 90.0)	50.0 (40.0, 60.0)	< 0.001

The clinical variables were averaged across five time points and then averaged across subjects (median, IQR)

**Fig. 2:**
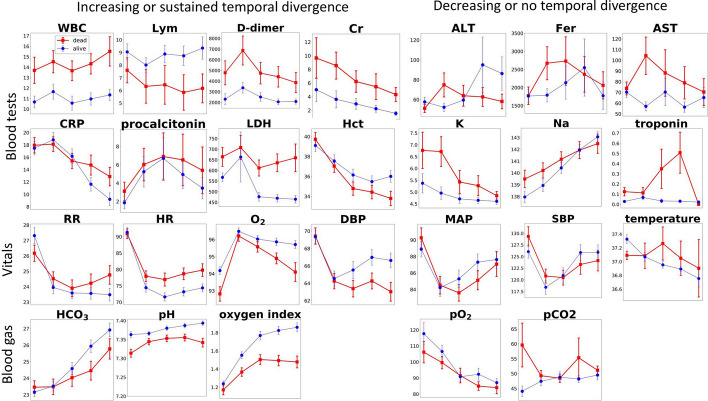
Longitudinal clinical variables from the day on mechanical ventilator. The variables are broadly grouped into those showed increasing or sustained temporal divergence and those showed decreasing or no temporal divergence. Red: death group. Blue: alive group. Error bars are SEM. See Table 1 for abbreviation definitions. Oxygen index is pO2:FiO2 where FiO2 is inspired oxygen fraction

Figure 2 shows the temporal evolution of the clinical variables during the first consecutive 5 days on mechanical ventilation. Among laboratory tests, white blood cell count (WBC), lymphocyte count (Lym), D-dimer, and creatinine (Cr) were consistently different across all 5 time points between groups. C-reactive protein (CRP), procalcitonin, and lactate dehydrogenase (LDH), and hematocrit (HCT) showed an increasing divergence between groups over time. Alanine aminotransferase (ALT), ferritin (Fer), aspartate aminotransferase (AST), potassium (K), sodium (Na), and troponin, did not differ between groups.

Among the vital signs, respiratory rate (RR), heart rate (HR), oxygen saturation (SpO_2_), and diastolic blood pressure (DBP) showed an increasing divergence between groups over time, whereas mean arterial pressure (MAP), systolic blood pressure (SBP), and temperature were similar between groups across time. Arterial pH, bicarbonate (HCO_3_), and oxygen index showed an increasing divergence between groups, whereas arterial pO_2_ and pCO_2_ did not.

### Predicting mortality

Predictive models of mortality were constructed using pCXR data alone, non-imaging data alone, and both combined in separate models utilizing data from day 1, day 5, day 1–3, day 3–5, or day 1–5 on IMV. To predict the binary outcome of mortality, prediction performance by ROC analysis was performed on the validation dataset (Table 2). AUCs from 5 consecutive day data were generally higher than those from 3 consecutive day data, which in turn were generally higher than those from single time-point data. AUCs were generally higher for models including both pCXR and non-imaging data, as opposed to models based on either one exclusively. Overall, prediction performance using 5-consecutive-day data was stronger (AUC = 0.80–0.87) than those using 3-consecutive-day data (AUC = 0.71–0.81), which were stronger than those using one-day data (AUC = 0.67–0.74). Prediction performance using day 5 data (one day of data) was better than that using day 1 data one day of data. Similarly, prediction performance using day 3–5 was better than that using day 1–3 data. The best predictive model was obtained using 5 consecutive days of combined pCXR and non-imaging clinical data, yielding an accuracy of 85 ± 3.5% (95% CI) and AUC of 0.87 ± 0.05 (95% CI). Precision, recall, and F1 score showed similar performance trends. By Delong’s statistical test for AUC differences, the AUC for the model using a combination of CXR and non-imaging data for days 1–5 was significantly different from AUC of day1 CXR (p = 0.02), day1 non-imaging (p = 0.03), day1 CXR + non-imaging (p = 0.02), and day1-3 CXR (p = 0.01). No other comparisons reached statistical significance.

**Table 2:** Performance metrics of models in predicting mortality using CXR data alone, non-imaging data alone, and their combination for 1, 3 and 5 days on mechanical ventilator

		AUC	Accuracy	Precision	Recall	F1 score
Day 1	CXR	0.67 (0.18)	0.75 (0.08)	0.58 (0.18)	0.56 (0.13)	0.55 (0.10)
	Non-imaging variables	0.69 (0.06)	0.74 (0.06)	0.59 (0.07)	0.64 (0.32)	0.57 (0.18)
	CXR + non-imaging variables	0.70 (0.14)	0.78 (0.08)	0.79 (0.19)	0.56 (0.26)	0.60 (0.22)
Day 5	CXR	0.70 (0.09)	0.73 (0.03)	0.62 (0.01)	0.56 (0.21)	0.57 (0.13)
	Non-imaging variables	0.69 (0.05)	0.73 (0.05)	0.66 (0.07)	0.50 (0.15)	0.55 (0.09)
	CXR + non-imaging variables	0.74 (0.04)	0.78 (0.02)	0.69 (0.09)	0.63 (0.08)	0.65 (0.07)
Day 1–3	CXR	0.71 (0.04)	0.76 (0.04)	0.69 (0.10)	0.67 (0.12)	0.67 (0.06)
	Non-imaging variables	0.77 (0.06)	0.80 (0.04)	0.85 (0.14)	0.42 (0.23)	0.51 (0.21)
	CXR + non-imaging variables	0.78 (0.05)	0.80 (0.04)	0.87 (0.15)	0.56 (0.20)	0.65 (0.13)
Day 3–5	CXR	0.77 (0.07)	0.78 (0.03)	0.81 (0.04)	0.61 (0.10)	0.69 (0.07)
	Non-imaging variables	0.78 (0.04)	0.78 (0.04)	0.69 (0.06)	0.61 (0.05)	0.65 (0.05)
	CXR + non-imaging variables	0.81 (0.00)	0.80 (0.03)	0.83 (0.12)	0.61 (0.19)	0.67 (0.11)
Day 1–5	CXR	0.80 (0.05)	0.80 (0.03)	0.77 (0.14)	0.70 (0.16)	0.71 (0.05)
	Non-imaging variables	0.83 (0.03)	0.82 (0.02)	0.82 (0.11)	0.69 (0.09)	0.74 (0.05)
	CXR + non-imaging variables	0.87 (0.06)	0.85 (0.04)	0.80 (0.04)	0.68 (0.08)	0.74 (0.06)

Values in parentheses are standard deviations

### Predicting duration of IMV

**Fig. 3:**
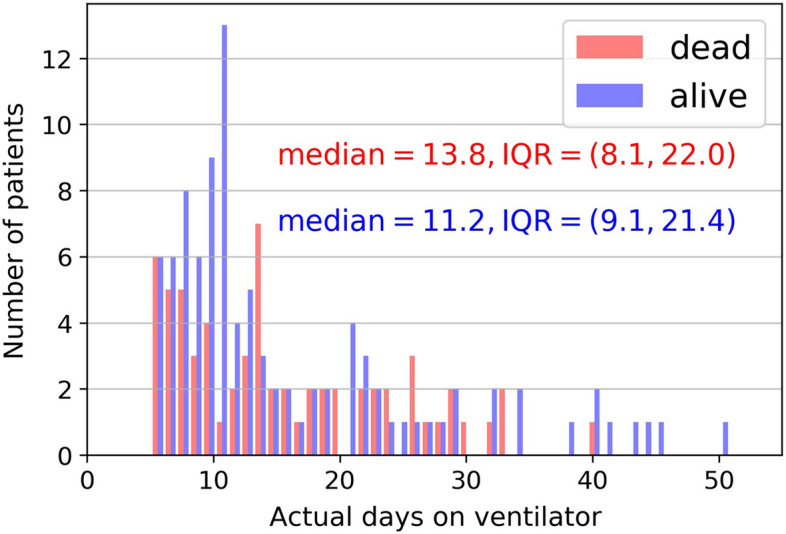
Histogram of days on IMV of the dead and alive group. Note that to maintain the same cohort that had at least 5 consecutive day data, patients with < 5 days of data were excluded from subsequent analysis

Figure 3 shows the histogram of days on IMV for the non-survivor and survivor groups. The number of days on IMV was not significantly different between non-survivors (median 13.8 [IQR:8.1, 22.0]) and survivors (11.2 days [IQR = 9.1, 21.4], *p* = 0.7). Although median durations on IMV did not differ between groups, there were more survivors than non-survival around 10 days of IMV. The number of days on IMV ranged from 1 to 51.

**Fig. 4:**
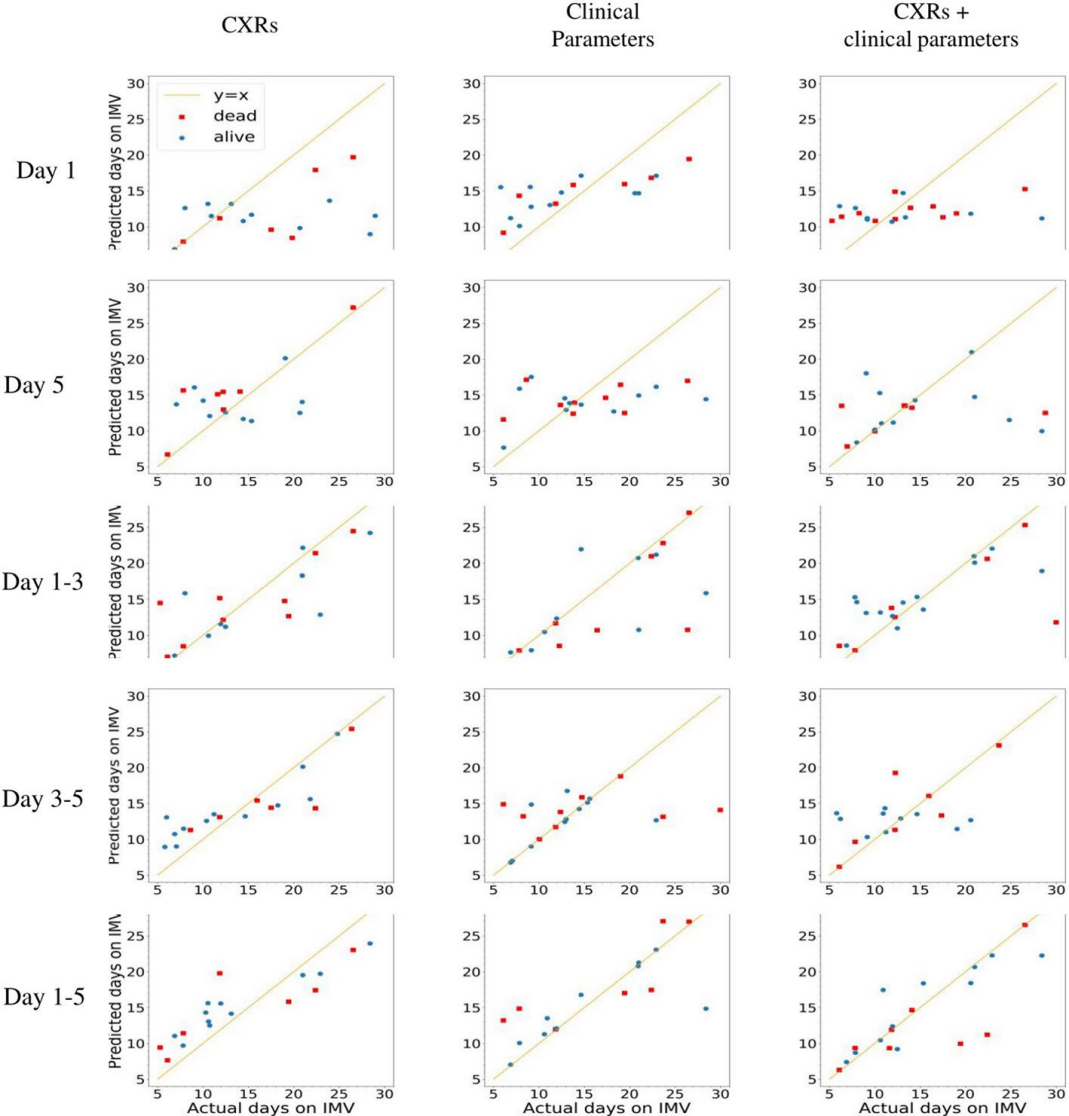
Correlation plots of predicted and actual duration on IMV of the validation dataset (onefold in fivefold cross-validation) for nine experiments. Yellow lines are lines of linearity

Predictive models of the duration of IMV were constructed in similar configurations. Correlation analysis of predicted and actual duration on IMV for all 15 experiments was performed on the validation dataset. The correlation plots, the quantitative values of slopes, intercepts, correlation coefficients, p values, mean absolute error (MAE), and root mean squared error (RMSE) of the prediction are summarized in Fig. 4 and Table 3. Correlations were stronger for the combined imaging and non-imaging clinical data than pCXR data alone or non-imaging clinical data alone (except the day 1–5 pCXR which showed the strongest (*R*^2^ = 0.8) correlation). Correlations for non-imaging data alone were slightly stronger than those for pCXR data alone. With respect to MAE, the best prediction performance was obtained using both pCXR and non-imaging clinical data over five consecutive days, which predicted the duration on the ventilator to within 2.56 ± 0.21 (95% CI) days.

**Table 3:** Performance metrics of models predicting days on ventilator using pCXR alone, non-imaging data alone and their combination

		Slope	Intercept	*R*^2^	*p*	MAE
Day 1	CXR	0.19 (0.08)	8.5 (1.1)	0.18 (0.09)	0.059	5.30 (0.42)
	Non-imaging variables	0.27 (0.10)	11.0 (1.3)	0.50 (0.14)	< 0.001	4.67 (0.33)
	CXR + non-imaging variables	0.07 (0.02)	11.0 (2.1)	0.11 (0.04)	0.120	4.54 (0.36)
Day 5	CXR	0.32 (0.08)	8.03 (1.9)	0.32 (0.11)	0.002	5.01 (0.44)
	Non-imaging variables	0.40 (0.12)	7.94 (1.7)	0.25 (0.19)	< 0.001	4.88 (0.38)
	CXR + non-imaging variables	0.41 (0.15)	6.73 (2.1)	0.37 (0.20)	0.008	4.21 (0.56)
Day 1–3	CXRs	0.51 (0.13)	6.49 (1.3)	0.58 (0.11)	< 0.001	3.41 (0.32)
	Non-imaging variables	0.62 (0.18)	3.76 (1.1)	0.54 (0.12)	< 0.001	3.13 (0.35)
	CXR + non-imaging variables	0.47 (0.12)	7.69 (1.2)	0.53 (0.11)	< 0.001	2.96 (0.33)
Day 3–5	CXRs	0.57 (0.16)	8.85 (1.6)	0.52 (0.12)	< 0.001	3.14 (0.57)
	Non-imaging variables	0.62 (0.17)	5.43 (1.1)	0.50 (0.15)	< 0.001	3.11 (0.32)
	CXR + non-imaging variables	0.59 (0.17)	6.79 (1.3)	0.51 (0.13)	< 0.001	3.05 (0.41)
Day 1–5	CXRs	0.60 (0.13)	6.55 (1.1)	0.80 (0.18)	< 0.001	3.11 (0.25)
	Non-imaging variables	0.62 (0.12)	6.26 (1.0)	0.69 (0.18)	< 0.001	2.88 (0.25)
	CXR + non-imaging variables	0.69 (0.10)	3.52 (0.7)	0.66 (0.15)	< 0.001	2.56 (0.24)

Values in parentheses are standard deviations

*MAE* mean absolute error

## Discussion

The temporal characteristics of clinical variables and pCXR from the first five days on IMV offer important insights into disease progression. There have been no studies to date that systematically investigated the temporal evolution clinical variables and pCXR during IMV. Deep learning predictions of in-hospital mortality and duration on IMV using combined imaging and non-imaging data outperformed either one alone. Deep-learning predictions using longitudinal data outperformed those using single time-point data. The best prediction performance was obtained using both pCXR and non-imaging clinical data over 5 consecutive days, yielding an accuracy of 85% for predicting mortality, and an MAE of 2.56 days for predicting the duration on IMV.

The relatively poor prediction performance using only *single-time-point* pCXR data alone, non-imaging data alone, and their combination is not unexpected as patients who were admitted to the ICU are in various stages of the disease severity. Data at a single time point are less accurate in predicting mortality or the continued need for IMV. Although a few studies have used single-point admission data to predict clinical outcomes in COVID-19, there is currently no consensus as to which clinical variables are most predictive of eventual clinical outcomes [29, 31–35]. Using 5-day instead of 1-day pCXR data, prediction of mortality improved AUC from 0.69 to 0.80 (21% improvement), and prediction of duration on IMV markedly improved MAE from 5.3 days to 3.11 days (41% improvement).

Prediction performances by pCXR and non-imaging data are similar, with non-imaging data models performing slightly better. It is also interesting to note that prediction with day 1–5 pCXR performed better than all day 1 and day 1–3 clinical variables, suggesting that longitudinal pCXR may have prognostic value. pCXR has limited utility in COVID-19 management to date and the majority of clinical management decisions currently rely on laboratory tests, clinical and vital signs. This is due in part to the incompletely understood pCXR disease progression, and the lack of standardized quantitative measures of disease severity from radiology reports to track changes longitudinally. pCXR can offer better insight into prognosis and expected duration of treatment than previously known. This data can then be used to anticipate future ICU bed needs and counsel patients and family members regarding the upcoming clinical course and the likelihood of survival.

Predictive models using a combination of non-imaging and pCXR data outperformed either alone, as indicated by AUC and correlation analysis. Predictive models using longitudinal data outperformed those using single time-point data. Moreover, the error bars were smaller with day 1–5 combined data compared to day 1–3 and day 1 data. For data closer to the outcome, prediction performance using day 1–5 data was better than that using day 1 data. Similarly, prediction performance using day 3–5 was better than that using day 1–3 data. AUC appeared to further increase with additional time points in this cohort and additional studies (i.e., including data of all 7 days) are needed. Nonetheless, these findings together suggest that deep learning of longitudinal pCXR and clinical variables have prognostic value. There have been no studies to date that used deep learning to predict mortality and duration on IMV with which to compare our findings.

Although previous studies have identified a few clinical variables [1, 2, 29, 36–41] and pCXR [14, 22–26, 28, 42] associated with COVID-19 infection, only a few studies have attempted to develop methods to predict mortality and disease severity. Essentially all studies today employed a single time point, usually at admission, and not longitudinal data. Our study is novel because it included longitudinal clinical variables data and pCXR.

This study had several limitations. This was a retrospective study that entails potential residual confounding and selection bias. This model only works with 5 consecutive days of data (from IMV admission). It has not been tested for any 5 days of data. The sample size is relatively small from a single hospital, which may not generalize to other settings. Using a larger sample size would be helpful, but the sample size of IMV COVID-19 patients was inherently limiting. Prospective study and testing on multiple institutional data with large sample sizes are needed to attain generalizability. COVID-19 pandemic circumstances are unusual and evolving, depending on the location and timing of the outbreak. The decision to place patients on mechanical ventilators and mortality rates may depend on an individual hospital’s patient load, practice, and available resources. Access to mechanical ventilators in this cohort was not a limiting factor in our hospital. It is conceivable that our model might not work for patients from other hospitals because their patients were more or less severe, among others. This study did not account for treatment effects. Patients were treated per standard of care and complete treatment details were not available on this dataset. It is generally challenging to account for treatment effects and different patients could respond differently to the same treatments. Our model did not account for other important variables such as types of symptoms, duration of symptoms, and co-infection, or other in-hospital developed medical issues that led to IMV. We narrowly focused on how CXR and clinical variables obtained from day 1–5 consecutive predict mortality. Our model also might not work on data from the second COVID-19 wave. This is not because the model is wrong, but rather we believe that it is necessary to retrain the models with local data. To date, it is generally not trivial for hospitals to share clinical COVID-19 data because of lack of infrastructure to do so seamlessly or concerns about patient data privacy, among others. Future studies may investigate predictions using radiologist clinical pCXR scores, and whether pre-intubation CXR predicts the need for IMV.

## Conclusion

Deep learning applied to pCXR and clinical variables reveals that serial data markedly improve the prediction of mortality and length of time on mechanical ventilation. In principle, pCXR could also be used to monitor disease progression and treatment response, optimize mechanical ventilator settings, determine when to safely extubate, and predict clinical outcomes in COVID-19 patients. However, the temporal progression of COVID-19 lung infection on pCXR is not completely understood [11]. These approaches, pending further confirmation, may facilitate prognosis, care planning, and resource allocation early in the course of critical care.

## Methods

### Study design, population, and data collection

This retrospective study was approved by our Institutional Review Board with an exemption of informed consent. Our study followed the Strengthening of Reporting of Observational Studies in Epidemiology (STROBE) reporting guidelines for cross-sectional studies (http://www.equator-network.org/reporting-guidelines/strobe/).

### Input variables

The input variables include serial pCXR, demographic information (age, sex, ethnicity, and race), chronic comorbidities (smoking, diabetes, hypertension, asthma, chronic obstructive pulmonary disease, coronary artery disease, heart failure, cancer, immunosuppression, and chronic kidney disease), serial vital signs (heart rate, respiratory rate, pulse oxygen saturation, systolic blood pressure, temperature, diastolic blood pressure, mean arterial pressure, FiO2, pCO2, HCO3, pH, pO2, hematocrit, potassium, and sodium), and serial laboratory tests (C-reactive protein, D-dimer, ferritin, lactate dehydrogenase, lymphocyte count, procalcitonin, alanine aminotransferase, brain natriuretic peptide, troponin, white blood cell, and aspartate aminotransferase).

### Statistical methods

Statistical analyses were performed in SPSS v26 (IBM, Armonk, NY). Categorical variables are presented as frequencies and percentages, and the comparison between groups was made using *χ*^2^ tests. Continuous variables are presented as medians and interquartile ranges (IQR), and the comparison between groups was made using Mann–Whitney* U* tests.

#### Outcome measures

The two outcomes were in-hospital mortality and the duration of IMV in days (i.e., how much time the patient needs to be on IMV). A total of 15 predictions were made as follows: Patient mortality and duration on IMV were predicted using either: *a)* data from the first day of IMV only (day 1 data); *b)* data from the fifth day of IMV only (day 5 data); *c)* data from the first three consecutive days of IMV (day 1–3 data); *d)* data from consecutive days 3–5 of IMV (day 3–5 data); or *e*) data from the first five consecutive days of IMV (day 1–5 data). Predictions were made using: *i)* pCXR data alone, *ii)* non-imaging data alone, and *iii)* both pCXR and non-imaging data.

#### Deep-learning architecture

**Fig. 5:**
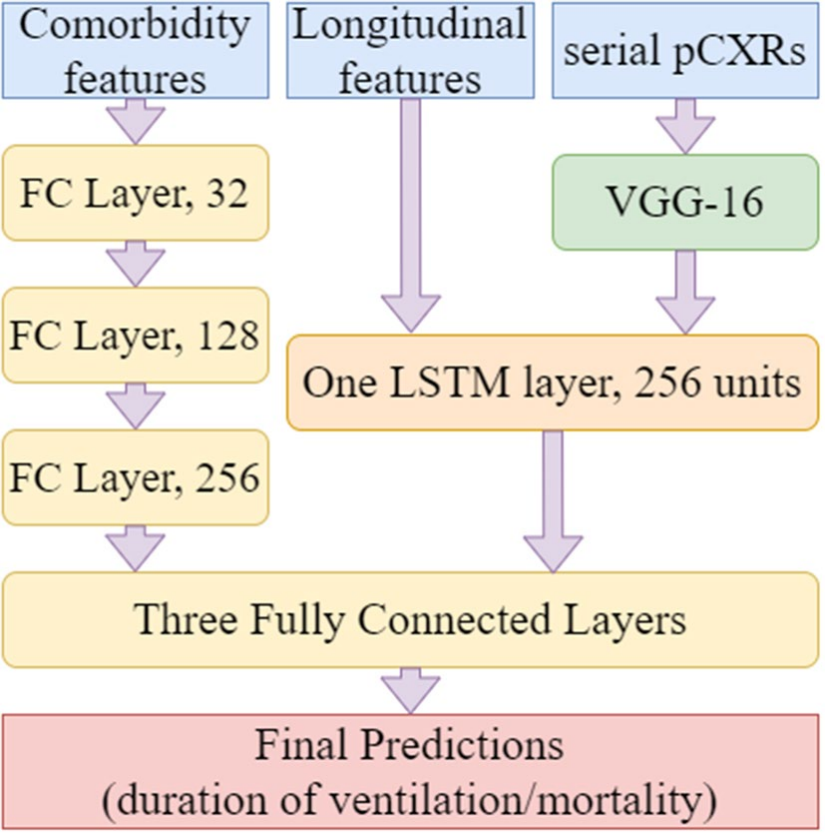
Architecture of the deep learning algorithm

The architecture of the deep learning algorithm (Fig. 5) consists of three main inputs: serial pCXRs, serial non-imaging features, and demographics/comorbidities. A 2D convolutional neural network (CNN) module designed to capture image patterns from pCXRs was based on VGG-16, a classical CNN architecture that has been widely proven to be effective [43]. The 2D CNN module consists of five convolutional blocks: the first two blocks have two convolutional layers while the last three have three convolutional layers. The last convolutional layer in each block is set to a stride of two to replace the maxpooling in the original VGG architecture, which is proven to be better in the ability of non-linear fitting [44]. In order to balance the computing burden and effectiveness of the system, the number of channels is reduced from 64-128-256-512-512 in the original VGG network to 16-32-64–128-128 in our system. The activation function ‘ReLU’ is followed by each convolutional layer to introduce non-linearity into the system. Batch normalization layers are deployed as well to ensure the stability of the training and reduce the risk of overfitting. After normalization, longitudinal features including serial vital signs and serial laboratory tests are then concatenated with the image patterns extracted from pCXRs before being fed into one long-short time memory (LSTM) layer, which is a deep learning technique aiming at processing time-series data. LSTM layer is a type of recurrent neural network (RNN) layer. Compared to traditional RNN layers, LSTM can control memory over time and the flow of information into and out of the layer through the use of three “gates”, the input, output, and forget gates [45]. SGD optimizer was used with a learning rate of 1e-4. Nesterov momentum was applied with momentum set as 0.9 to avoid local minima for loss. Categorical cross-entropy was used as the loss function to measure the difference between predicted results and ground truth. The training process lasted for 20 epochs. Inside of the LSTM layer, 200 hidden units are deployed, and the hyperbolic tangent (tanh) function is set as the activation function. After that, non-longitudinal features including demographic information and chronic comorbidities processed by three fully connected layers are concatenated with the output from LSTM and then all features extracted from three resources are fed into three more fully connected layers to make the final predictions. Between the last three fully connected layers, two dropout layers with a drop rate of 0.1 are deployed to prevent overfitting.

#### Performance evaluation

**Fig. 6:**
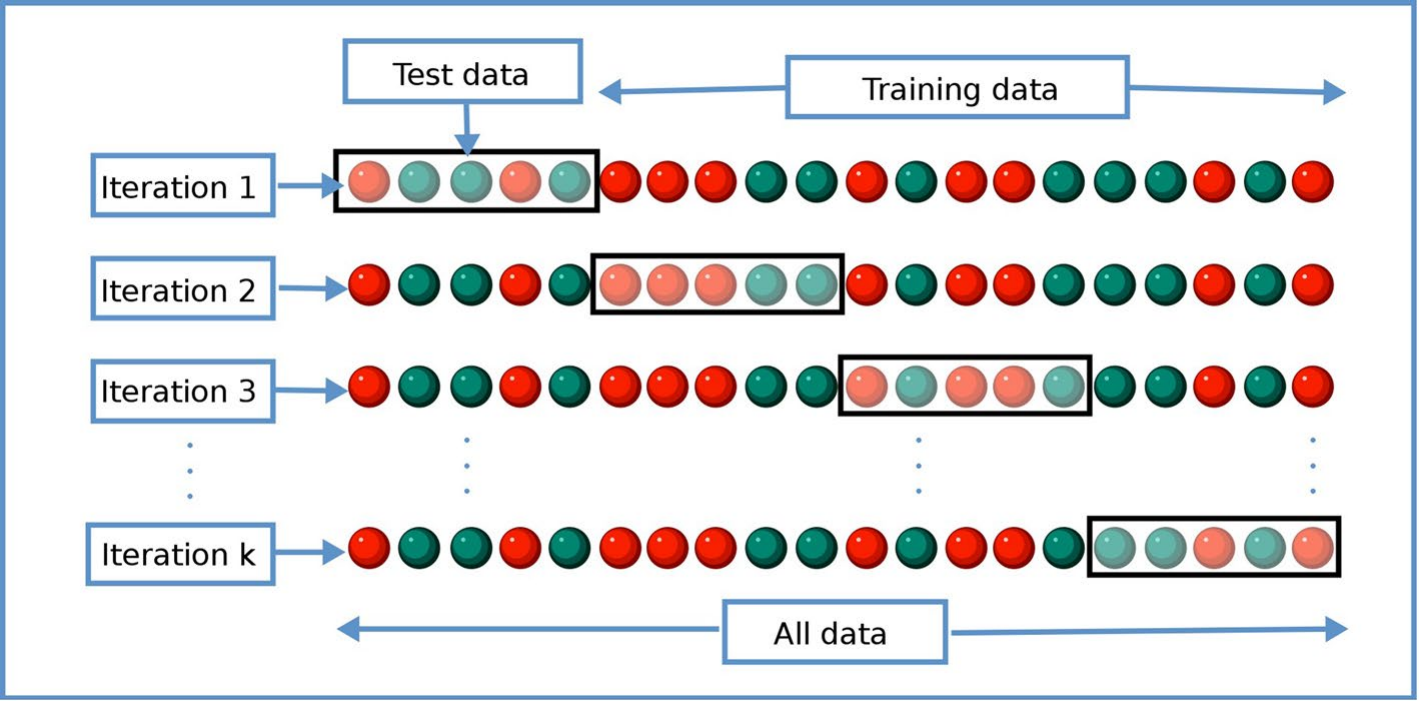
Diagram showing how data are divided for k-fold cross-validation

To predict mortality (binary variable), ROC analysis was employed with area under curve (AUC), accuracy, precision, recall, and F1 score. Results are reported in fivefold cross-validation. Figure 6 shows a diagram of the cross-validation process. In stratified fivefold cross-validation, the dataset was split into five subsets, each with the same sample size and with an equal ratio of samples from each outcome class. Four-fifths of the data were used to train the model while one-fifth of the data were held out for validation, creating an 80%:20% of training:validation split. This is repeated five times so each fold was used as the validation set once. The performance reported was the averaged of the five repeats. Each fold was evaluated using the same metrics: accuracy, AUC, specificity, and sensitivity. Individual values for each fold were not reported, rather an average value with standard deviation is shown in Tables 2 and 3. The internal validation hold set was treated as a testing set and was not touched in the training process. There was no external validation of data from another institution due to difficulty in obtaining such detailed data. DeLong statistical test was used to compare AUC differences between groups. A *p*-value < 0.05 was considered to be statistically significant. To predict duration on IMV (continuous variable), correlation analysis was employed. Slopes, intercepts, correlation coefficients, *p* values, and mean absolute errors (MAE) were calculated.

To minimize overfitting, we employed the following approaches: (1) ReLU was used as the activation function and batch normalization layers were deployed to minimize overfitting; (2) fivefold cross-validation was used; (3) regularization was used; (4) early stopping of the training was deployed when no improvements were seen for 10 epochs, and (5) only clinical variables that were relevant to predicting mortality from our previous studies were used.

## Data Availability

The datasets generated and analyzed during the current study are available from the corresponding author on reasonable request.

## Abbreviations

ALT: Alanine aminotransferase
AST: Aspartate aminotransferase
AUC: Area under the ROC curve
BNP: Brain natriuretic peptide
CAM: Class activation map
CI: Confidence interval
CNN: Convolutional neural network
COVID-19: Coronavirus disease 2019
CRP: C-reactive protein
CT: Computed tomography
ED: Emergency department
IMV: Invasive mechanical ventilation
IQR: Interquartile ranges
LDH: Lactate dehydrogenase
LSTM: Long-short time memory
MAE: Mean absolute error
ML: Machine learning
pCXR: Portable chest X-ray
ReLU: Rectified linear unit
RMSE: Root mean squared error
ROC: Receiver operating characteristic
RT-PCR: Real-time polymerase chain reaction
SARS-CoV-2: Severe acute respiratory syndrome coronavirus 2
SpO2: Pulse oxygen saturation
WBC: White blood cell
